# Advances in Lung Cancer Driver Genes Associated With Brain Metastasis

**DOI:** 10.3389/fonc.2020.606300

**Published:** 2021-01-18

**Authors:** Yalin Kang, Yu Jin, Qianxia Li, Xianglin Yuan

**Affiliations:** Department of Oncology, Tongji Hospital, Tongji Medical College, Huazhong University of Science and Technology, Wuhan, China

**Keywords:** brain metastasis, lung cancer, driver gene, receptor tyrosine kinase, epithelial-mesenchymal transition, colonization

## Abstract

Brain metastasis, one of the common complications of lung cancer, is an important cause of death in patients with advanced cancer, despite progress in treatment strategies. Lung cancers with positive driver genes have higher incidence and risk of brain metastases, suggesting that driver events associated with these genes might be biomarkers to detect and prevent disease progression. Common lung cancer driver genes mainly encode receptor tyrosine kinases (RTKs), which are important internal signal molecules that interact with external signals. RTKs and their downstream signal pathways are crucial for tumor cell survival, invasion, and colonization in the brain. In addition, new tumor driver genes, which also encode important molecules closely related to the RTK signaling pathway, have been found to be closely related to the brain metastases of lung cancer. In this article, we reviewed the relationship between lung cancer driver genes and brain metastasis, and summarized the mechanism of driver gene-associated pathways in brain metastasis. By understanding the molecular characteristics during brain metastasis, we can better stratify lung cancer patients and alert those at high risk of brain metastasis, which helps to promote individual therapy for lung cancer.

## Introduction

Lung cancer, accounting for 18.4% of the total cancer population, ranks first in cancer-associated mortality globally. Brain metastasis is one of the main causes of death in patients with lung cancer (1). With the advancement of cancer treatment strategies, cancer mortality has continued to decline since 1991. The decrease of mortality is particularly pronounced for lung cancer in recent years, which decreased by 5% per year (2013 to 2017) compared to 3% per year (2008 to 2013) in men, and in women by 4% per year compared to 2% per year (2). The prevalence of brain metastases in patients with advanced lung cancer is about 20~56%, accounting for 40~50% of all brain metastases (3–5). According to histopathology, lung cancer is classified into two types: non-small cell lung cancer (NSCLC) (85%) and small cell lung cancer (SCLC) (15%). SCLC has a single histological type and is highly aggressive. About 50% of patients presents with brain metastases at diagnosis and during treatment (6). At present, prophylactic cranial irradiation (PCI) can be used for SCLC, while for NSCLC, effective strategy to prevent brain metastases is still lacking (6). The RTOG 0214 trial of PCI in NSCLC showed that PCI reduces brain metastasis rates after 1 year (18 *vs.* 7.7%), but the overall survival is not improved (7). It revealed that identifying candidates who could benefit from PCI was difficult. Hence, for patients with NSCLC, it is important for clinician to detect the patient at high risk of brain metastasis.

Since Paget put forward the “seed-soil” hypothesis in 1889, theories such as cancer stem cells (CSCs), tumor microenvironment, and circulating tumor cells have been proposed successively, further supplementing the mechanism of tumor metastasis (8–12). In 2003, evolutionary geneticist Austin Burt proposed the concept of “gene drive,” thinking that cancer is a genetic disease, in which gene mutations eventually result in phenotype changes, leading to the occurrence of cancer (13). With the popularization of high-throughput sequencing technology, genetic cloning events have again caught people’s eye regarding tumorigenesis (14). Endogenous mutation process drives the occurrence of lung adenocarcinoma (15). Driver mutations can arise before and after subclonal diversification, and the subclonal mutations may be important for cancer progression (16, 17). By tracking the driving events of patients, researchers have found that genetic diversity is a determinant of patient outcome, and clonal evolution or chromosomal instability is suggested as a biomarker for detection and intervention of disease progression (18–21). Moreover, the Genotype-Tissue Expression project found that local genetic variation affects the expression level of most genes (22, 23). These findings suggested that driver genes could be used as biomarkers to predict tumor metastasis.

In lung cancer, patients with mutation of common driver genes (such as *EGFR* and *ALK*) can benefit from targeted therapy. Progress has also been achieved in research on rare mutations such as *ERBB2*, *MET*, *RET*, *ROS1*, and *PIK3CA*, and new inhibitors targeting the products of these genes are under development (24, 25). In recent years, several studies have found that driver genes have a predictive role in the occurrence of brain metastases (26–28). In this review, we summarized the current advances of lung cancer driver genes in the occurrence of brain metastasis and the related mechanisms, hoping to provide a general understanding for researchers and clinical doctors.

## Lung Cancer Driver Genes and Brain Metastasis

**Figure 1:**
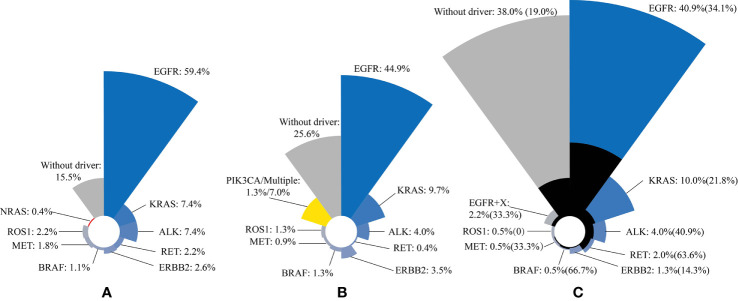
Three studies have shown that the most common type of lung cancer driver gene mutation is *EGFR* mutation, followed by *KRAS* mutation and *ALK* fusion mutation. The black area indicates the percentage of brain metastases in the total number of corresponding mutations. **(A)** n = 271; **(B)** n = 227; **(C)** n = 552. The incidence of brain metastases in patients with driver gene mutations is shown in brackets.

In a study of 271 patients with lung adenocarcinoma, 85% of patients have driver gene mutations (29) (**Table 1**, **Figure 1**). A recent study reported that 74.4% of lung adenocarcinoma patients have at least one druggable mutation detected (30) (**Table 1**, **Figure 1**). In another study of 552 NSCLC patients, mutation of driver gene is present in 62% of patients, and those with driver gene mutations are more prone to brain metastases (26) (33 *vs.* 19%; **Table 1**, **Figure 1**). The three studies suggested that the incidence of driver gene mutations in NSCLC patient is high, and lung cancer driver genes, such as *EGFR*, *ALK*, and *RET* are risk factors for brain metastasis in advanced NSCLC patients. Tomasini et al. previously proved that *EGFR* and *KRAS* mutations have a predictive role on brain metastasis incidence, recurrence, and outcome in NSCLC patients (27). Patil confirmed that brain metastasis is common in patients with *ROS1*-positive advanced NSCLC (31). New brain metastasis driver genes, such as *MYC*, *AXIN2*, and *NRG1*, have also been discovered (32–35). At present, researchers have mainly studied the incidence of common driver genes and the rate of brain metastasis in NSCLC patients. New driver genes, due to their low incidence, have not been studied yet, although they may be involved in the occurrence and development of brain metastasis (**Table 2**).

**Table 1:** The distribution of the main driver gene mutation types of patients with lung cancer.

Type	n = 271 (LUAD) Total (n, %)	n = 227 (LUAD) Total (n, %)	n = 552 (NSCLC) Total (n, %)	n = 153 BM (n, %^1^/%^2^)
No driven gene mutation	42 (15.5)	58 (25.6)	210 (38.0)	40 (26.1/19.0)
Driver gene mutation	229 (84.5)	169 (74.4)	342 (62.0)	113 (73.9/33.0)
*EGFR* mutation	161 (59.4)	102 (44.9)	226 (40.9)	77 (50.3/34.1)
*KRAS* mutation	20 (7.4)	22 (9.7)	55 (10.0)	12 (7.8/21.8)
*ALK* fusion mutation	20 (7.4)	9 (4.0)	22 (4.0)	9 (5.9/40.9)
*RET* fusion mutation	6 (2.2)	1 (0.4)	11 (2.0)	7 (4.6/63.6)
*ERBB2* mutation	7 (2.6)	8 (3.5)	7 (1.3)	1 (0.7/14.3)
*BRAF* mutation	3 (1.1)	3 (1.3)	3 (0.5)	2 (1.3/66.7)
*MET* mutation/amplification	5 (1.8)	2 (0.9)	3 (0.5)	1 (0.7/33.3)
*ROS-1* fusion mutation	6 (2.2)	3 (1.3)	3 (0.5)	0 (0.0/0.0)
*NRAS* mutation	1 (0.4)	–	–	–
*PIK3CA*/Multiple mutation	–	3/16 (1.3/7.0)	–	–
*EGFR* mutation +X^3^	–	–	12 (2.2)	4 (2.6/33.3)

^1^Number of brain metastasis/Total number of brain metastasis.

^2^Number of brain metastasis/Total number of the mutations or total number of no driver gene mutation.

^3^X genes included the mutations of ALK, KRAS, BRAF, ERBB2, and MET amplifications.

LUAD, lung adenocarcinoma; NSCLC, non-small cell lung cancer; BM, brain metastasis.

**Table 2:** Potential biomarkers in lung cancer driver genes and the targeted drugs in brain metastasis.

Gene	Mutation rate in NSCLC (%)	Ref	BM rate in NSCLC (with *vs.* without mutation) (%)	Ref	Drug (Treat BM or have intracranial activity)	Trial	Ref
*EGFR*	29.4 40.9 33.2–59.4	(28) (26) (29–31, 36)	31.4 *vs.* 19.7 34.1 *vs.* 19.0 27.6–52.9 ^1^	(28) (26) (31, 37)	Afatinib Erlotinib Icotinib AZD3759 YH25448	Clinical Clinical Clinical Preclinical Preclinical	(38) (39) (40) (41) (42)
*ALK*	4.0 4.0–19.9	(26) (29–31, 43)	40.9 *vs.* 19.0 23.8–58.4 ^1^	(26) (31, 37)	Alectinib Crizotinib Lorlatinib	Clinical Clinical Clinical	(44) (45, 46) (47, 48) NCT03052608
					PF-06463922	Preclinical	(49)
*ROS1*	0.5–5.7	(26, 29–31)	19.4–47.4 ^1^	(31, 50, 51)	Entrectinib Repotrectinib	Clinical Preclinical	(52, 53) (54)
*MET*	0.5–1.8	(26, 29, 30)	33.3 *vs.* 19.0 ^2^	(26)	Cabozantinib Tepotinib	Case report Case report	(55) (56)
*RET*	2.0 0.4–2.2	(26) (29, 30)	63.6 *vs.* 19.0 24.8–46.5 ^1^	(26) (57)	Selpercatinib Pralsetinib	Clinical Clinical	(58) NCT03037385 NCT04222972
*KRAS*	7.4–45.5	(26, 29, 30, 59)	21.8 *vs.* 19.0 16.8–28.4 ^1^	(26) (31, 60)	–	–	–
*BRAF*	0.5–3.5	(26, 29, 30, 61)	66.7 *vs.* 19.0 ^2^ 18.8 ^1^	(26) (31)	–	–	–
*ERBB2*	1.3–3.5	(26, 29, 30)	14.3 *vs.* 19.0 ^2^	(26)	–	–	–
*NRG1*	1.7	(62)	–	–	–	–	–
*PIK3CA*	1.3–4.2	(30, 63)	–	–	–	–	–

^1^There is no brain metastasis data from patients without driver gene mutations in the corresponding references.

^2^Total number of the mutations <10.

However, gene mutations in brain metastatic sites are inconsistent with the original lesions, and acquired mutations can occur during treatment, resulting in drug resistance and disease progression. For example, inconsistent mutation status of *KRAS* have been discovered between the primary and metastatic sites of lung adenocarcinoma, while the status of *EGFR* mutation is relatively consistent on the contrary (64). In Brastianos’s study, 86 matched brain metastases, primary tumors and normal tissues were sequenced with whole exome sequencing to check whether brain metastases had genetic changes different from the primary tumors. Changes were found only in brain metastases in about 53% of cases. Detected alteration was associated with the sensitivity to phosphatidylinositol 3 kinase (PI3K) pathway and epidermal growth factor receptor (EGFR) pathway inhibitors (65). Integrated genomic and transcriptomic analysis had identified crucial roles of EGFR signaling in brain metastasis (34). Furthermore, Paik and Wang H et al. also revealed a correlation of PI3K signaling with increased risks of brain metastasis in patients with NSCLC (32, 66). In Wang H’s study, mutations of *EGFR*, *KRAS*, and *ALK* are highly concordant between primary NSCLC and matched brain metastases, whereas discordance of PI3K signaling suggested the unique genomic evolution and oncogenic mechanisms of brain metastasis (32).

In addition, single nucleotide polymorphisms of driver genes are also closely related to the occurrence of lung cancer brain metastases. Li Q et al. proved for the first time that genetic variations in the transforming growth factor-β (TGF-β), PI3K/protein kinase B (AKT) pathways were associated with an increased risk of brain metastasis in NSCLC patients (67, 68). Recently, Xu Y et al. also proved that single nucleotide polymorphisms in the mammalian target of rapamycin complex 1 (mTORC1) signaling pathway are significantly associated with increased risk of brain metastasis (69). Activity of mTORC1/2 is higher in patients with lung cancer with brain metastases (70). Therefore, it is very necessary to clarify the status of lung cancer driver genes at the diagnosed with NSCLC, which can help predict the occurrence of brain metastasis. New driver genes may play a unique role in the mechanism of brain metastasis.

## Signaling Pathway in Lung Cancer Driver Gene

Lung cancer driver genes mainly include *EGFR*, *ERBB2*, *MET*, *RET*, *ALK*, and *ROS1*, all encoding genes for receptor tyrosine kinases (RTKs). Abnormal RTKs activation in tumors mainly includes acquired mutations, genome amplification, chromosome rearrangement, and autocrine activation, which lead to the imbalance of RTK signals and promote cell proliferation, metabolism, cytoskeleton remodeling, cell migration, and anti-apoptosis effects (71). RTKs form dimers by binding to their corresponding ligands or closely combining with members of the same family to stabilize and enhance downstream signaling pathways. The downstream signaling pathways of RTKs mainly include RAS (a GTPase)/RAF (a kinase)/mitogen-activated protein kinase (MAPK) and PI3K/AKT/mTOR (72–74). Other genes encoding important molecules in these pathways are also lung cancer driver genes. Li D et al. reported that driver gene mutations can occur within tyrosine kinase domains and genetic alterations frequently occurs in genes of the MAPK signaling, WNT signaling and mTOR pathways in patients with lung adenocarcinoma (75).

Classical driver genes can also interact with other pathways, which have been shown to be activated in lung cancer. Proteomic data have uncovered an interdependence of PI3K and signal transducer and activator of transcription 3 (STAT3) (76). Tyrosine-759, located in Janus kinase (JAK), acts as a docking site for the adaptor molecule SHP2, which is crucial for the initiation of the PI3K and MAPK pathway (77). Govindan et al. proved that JAK/STAT pathway is significantly altered in patients with lung cancer (78). RAS can also cross-link with the WNT/β-catenin pathway to promote tumor invasion (79). In addition, the downstream pathways of RTKs can also act synergistically with TGF-β receptors. TGF-β receptors levels can differentially affect the activation of the MAPK pathway (80). In a transgenic mouse model of *KRAS*-induced lung cancer, invasive adenocarcinoma is modeled by the loss of the TGF-β receptors (81). Interfering with these pathways can suppress lung cancer with positive driver genes. For example, preclinical findings have identified that inhibition of the interleukin-6 (IL-6)/STAT3 pathway can also inhibit tumor growth with EGFR mutation in NSCLC and suppress KRAS-driven lung adenocarcinoma (82–85). Mohrherr et al. proved that JAK/STAT pathway inhibitors can attenuate the progression of lung cancer driven by *KRAS* in preclinical models (86). In addition, TGF-β receptor inhibitors may be an effective therapy in a subset of *KRAS*-mutant patients with NSCLC (87). Molecules in these pathways can be considered as potential biomarkers in preventing lung cancer driver gene-associated brain metastasis.

## Mechanism of Lung Cancer Driver Genes in Brain Metastasis

Mutations introduced during primary tumor cell growth can result in clonal heterogeneity. Vogelstein put forward four types of genetic heterogeneity in tumors: intratumoral, intermetastatic, intrametastatic, and interpatient (88). Intratumoral heterogeneity provides the seeds for intermetastastic heterogeneity. Intratumoral heterogeneity mediated through chromosome instability is associated with an increased risk of recurrence or death in NSCLC and driven metastasis (19, 20, 89). Chromosome instability is increased in brain metastases and is a driver of metastasis (65, 90). Intermetastatic heterogeneity supports the idea that the genetic alterations required for metastasis are present before metastasis actually occurs (91). The founder clones are initiating events for lung cancer and other mutations are acquired later and perhaps are important for tumor progression (78). The outgrowth of distal colonizing cells necessitates further selection from subsequent genetic heterogeneity (92).

**Figure 2:**
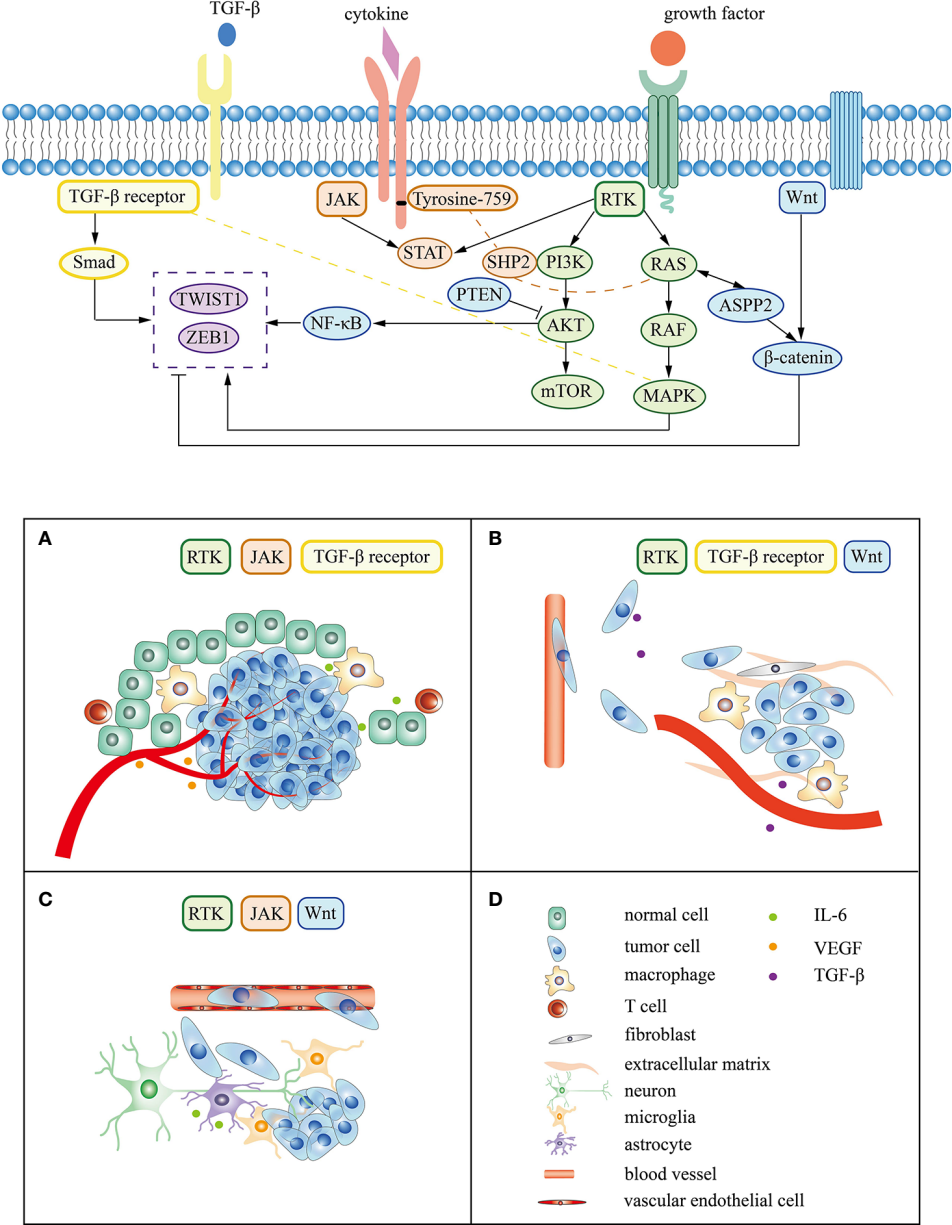
Signaling pathway and mechanism related to lung cancer driver gene in brain metastasis. **(A–C)** represent the tumor cell survival, EMT, and colonization respectively. **(D)** corresponds to the cell or molecule in **(A–C)**.

During the process of brain metastasis, abnormal genes drive tumor cells to escape normal regulatory mechanisms and change the microenvironment of lung cancer tumors through various signals, which continues to enhance tumor invasiveness, induce epithelial-mesenchymal transition (EMT) and accelerate vascular invasion (93). Metastatic tumor cells are arrest at vascular branch points, early extravasation, persistent close contacts to microvessels, and perivascular growth (94). Tumor cells cross the blood-brain barrier mediated by specific molecules and turn into dormancy/quiescence, laying the foundation for growth after several months or even longer (92, 95). Tissue remodeling creates a tumor microenvironment, affecting the homeostasis of the central nervous system and promoting tumor metastasis and growth (96). The following content mainly focuses on the mechanism of lung cancer driver genes and associated signaling pathway in the three main steps of brain metastasis (**Figure 2**).

### Promote Tumor Cell Survival

Phenotypic and functional heterogeneity arise among cancer cells within the same tumor. Comparing with cancer cells without driver gene mutation, those with a mutation, such as *EGFR*, *ERBB2*, *TGFbR2*, *MET*, *RAS*, *RAF*, *PIK3CA*, and *PTEN* genes, can proliferate under limiting nutrient concentrations (88). Cells with driver gene mutations will have a selective growth advantage than others. For instance, mutations in *KRAS* or *BRAF* genes confer on cancer cells the ability to grow in lower glucose concentrations (97, 98). Some of driver genes encode RTKs to receive the growth factor signal, whereas others are signal transducers of RTK-related pathways (99). After *ROS1* rearrangement, the extra-membrane part of the expressed protein is lost, leaving only the activated intra-membrane part, which fuses with other proteins and continuously transmits signals of growth and proliferation (100). Inhibition of these receptors or signals can interfere with cell growth and promote apoptosis. Inhibiting of EGFR activity increases apoptosis (101). Govindan et al. supposed that it is likely that driver gene mutations, such as *EGFR* and *KRAS*, are initiating events for lung cancer (78).

In cancer genome landscapes, driver genes regulate cell survival by the RAS/MAPK pathway, PI3K pathway, STAT pathway, and TGF-β pathway (88). Lysophosphatidylcholine acyltransferase 1 can up-regulate the PI3K/AKT pathway and promote *EGFR* mutation lung adenocarcinoma cell proliferation, invasion, and brain metastasis (102). Targeting lonidamine to mitochondria can inhibit AKT/mTOR signal, induce autophagic death of lung cancer cells with *KRAS* mutation and block tumorigenesis and brain metastasis (103). *PTEN* is an important gene that negatively regulates the AKT signaling pathway. Mutations in *PTEN* may have strong tumor-growth-promoting capability (75). In mouse models, tracheal epithelial cells lacking *PTEN* produce spontaneous tumors (104, 105). Abnormal activation of the above tumor driver genes leads to growth dominance and immortalization of lung cancer cells, opening the first step of tumor metastasis.

In the tumor microenvironment, driver gene-associated pathways are also involved in the formation of tumor immunosuppressive microenvironment, which is more conducive to the survival of tumor cells. In preclinical study, tumor cells use PI3K-hypoxia-inducible factor 1α axis to polarize macrophages into tumor-associated macrophages (TAMs), which produce IL-6 after engulfing particles released by tumor cells (106, 107). TAMs polarize towards M2 type through the IL-6/STAT3 signaling pathway to promote tumor metastasis and rejects immune cells from penetrating (108–110). Wu SY et al. proved that M2 macrophages are closely related to brain metastasis of lung cancer (111). On the other hand, by regulating mTOR, TAMs block normal glycolysis, induce excessive angiogenesis, and form abnormal blood vessels (112). These signals regulate the microenvironment of the primary tumor to escape from the immune system, and to create a microenvironment suitable for tumor growth and invasion. Moreover, activation of the EGFR pathway increases the production of tumor-derived vascular endothelial growth factor (VEGF), which acts on endothelial cells in a paracrine manner to promote angiogenesis (113). When driver gene is mutant or the coding molecule is activated, the above situation will be more likely to happen.

### Promote Epithelial-Mesenchymal Transition

EMT is a temporary and reversible process characterized by epithelial cell dedifferentiation and migration to a distance site (114). Bakhoum et al. found that metastatic tumors contain a large number of differentially upregulated EMT- and inflammation-related genes (90). Markers of EMT, including E-cadherins and N-cadherin, can be used as biomarkers to predict brain metastasis (115, 116). Various internal signals (such as gene mutations) and external signals (such as growth factor signals) play an important role in this process (117). Yousefi et al. put forward CSCs may originate from somatic mutations of normal tissue stem cells or may dedifferentiate from cancer cells *via* EMT (118). In the absence of driver gene mutations, RTK, TGF-β, and WNT pathways play an important role in EMT *via* activating transcription factors, such as twist family bHLH transcription factor 1 (TWIST1) and zinc finger E-box binding homeobox 1 (ZEB1) protein (118, 119). These signal pathways interact with each other to promote EMT (120–124).

As crucial internal signals, driver gene mutations give tumor cells stronger capability of EMT. For instance, driver gene *RAS* is closely related to EMT, and TWIST promotes tumor initiation and progression *in vivo* only after interaction with activated *RAS* (125). Activation of RAS can stimulate apoptosis-stimulating protein of p53 2 (ASPP2) and β-catenin to translocate from the cell junction to the cytoplasm and nucleus, reducing the formation of ASPP2-β-catenin complex, leading to EMT of tumor cells (79). In addition, mutation of TGF-β receptor can lead to loss of cytostatic effects of TGF-β. Tumor cells in the absence of cytostatic response may undergo EMT in response to TGF-β, which helps to escape the immunosuppressive environment and induce angiogenesis as well as systemic spread in 3D Tissue Culture (126, 127).

Therefore, many molecules can affect the occurrence and development of tumors by promoting or interfering with EMT. TAM induces EMT through IL-6-mediated WNT pathway to promote the invasion of lung cancer cells (128). MicroRNA-330-3p and Insulin-like growth factor binding protein-3 affect EMT by regulating the TGF-β/Smad signaling pathway, thereby promoting brain metastasis in NSCLC (129, 130). Programmed death ligand-1 may induce EMT by activating the TGF-β/Smad signaling pathway, and this process contributes to the primary resistance of *EGFR*-mutant NSCLC cells to TKIs (131, 132). ASPP2 can stabilize the β-catenin–E-cadherin complex and prevent β-catenin from transactivating ZEB1 to limit the aggressiveness of RAS and inhibit tumor metastasis *in vivo* (133, 134). Another study suggested that apoptotic lung cancer cells can increase the level of phosphatase and tensin homolog (PTEN) in exosomes from TAMs, which results in reduction of ZEB1 and inhibition of EMT (135).

### Affect the Colonization of Metastatic Tumor Cells Into the Brain

The PI3K/AKT, JAK/STST, and WNT signaling pathways are involved in vessel penetration and colonization of tumor cell to the brain (136). Attenuated WNT signaling is associated with the dormancy/quiescence of tumor cells in metastases (95). Ma SC et al. proved *in vitro* experiments that Claudin-5 regulates permeability of the blood-brain barrier by changing the proliferation, migration, and adhesion of brain microvascular endothelial cells, which resulted in decreased brain metastases from lung cancer (136). In preclinical study of breast cancer, heparin binding EGF, ligand of EGFR, can enhance the adhesion between tumor cells and brain endothelial cells, and help tumor cells penetrate the blood-brain barrier in breast cancer (92). Cathepsin S attenuates EGF-mediated EGFR degradation, which regulates EGFR signaling (137). Cathepsin S produced by tumor cells promotes tumor cell extravasation by accelerating the proteolysis of adhesion molecules between endothelial cells (138).

Tumor cells that enter the brain microenvironment interact with the original “residents” (mainly microglia and astrocytes), and grow autonomously in brain tissue through tumor-specific signaling pathways. Tumor cells interact with microglia and affect angiogenesis and survival through activation of STAT3 pathway in microglia (139). In multiple models of tumor metastasis, TAMs activate JAK/STAT signals to reverse EMT and promote metastatic colonization (140). Moreover, Chen Q et al. found that gap junctions between lung cancer cell and astrocyte triggers STAT1 survival signals *in vivo* and *in vitro* (141). Activated astrocytes produce IL-6, which in turn promotes lung cancer cell proliferation in Seike’s study (142). In brain metastases, astrocytes and tumor cells transduce bidirectional signals through endothelin and its receptors, as well as the AKT pathway, which produces chemotherapy protection (143). Unfortunately, this result is mainly verified in breast cancer cell lines.

However, despite the fact that driver genes are related to brain metastasis and driver gene-associated signaling pathways play a key role in colonization, there are still very few such preclinical studies on why lung cancer with driver gene mutation is more likely to develop brain metastasis than those without. At present, studies have focused on the relationship between patients with positive driver genes and the occurrence of brain metastasis, as well as the molecular mechanism of brain metastasis is not clear in patients with driver gene mutation. Preclinical models only center on the use of driver gene mutations in cell lines or animal models. For example, Nguyen demonstrated earlier that the WNT signaling pathway can enhance the ability of lung adenocarcinoma cells with *KAS* or *EGFR* mutation to colonize the brain (144). Adaptive loss of *PTEN* of breast cancer cells in brain metastasis, which was silenced by astrocyte-derived miRNAs, leads to increased secretion of chemokine (C-C motif) ligand 2, recruitment of myeloid cells, promotion of cell proliferation, and reduced apoptosis, which further enhances the growth of tumor cells in metastatic sites (104).

Why do tumor cells choose to “settled” in the brain? A reasonable explanation may be that the tumor cells that successfully grow, proliferate, and eventually form brain metastases have specific adaptations to the brain microenvironment. Transcriptome data of microarray hybridization showed that metastatic tumor cells are reprogrammed in the brain microenvironment to obtain neuronal cell characteristics (145). There is a similar situation in the lung cancer bone metastasis model. Tumor cells can acquire the characteristics of the metastatic microenvironment, which known as osteomimicry (146). Furthermore, residents in pre-metastasis microenvironment remodel the soil to promote seed growth in breast cancer lung metastasis model (147). In addition to the specific adaptations, tumor cells will also choose a more favorable microenvironment. Saunus’s research found that *EGFR*, *ERBB2*, and *ERBB3* transcripts were abundantly expressed in lung cancer brain metastases, and *ERBB3* transcript abundance correlated with its oncogenic partner *ERBB2* (34). However, expression of neuregulin 1, which is the ligand for erb-b2 receptor tyrosine kinase 3, is very low in tumor cells and rich in brain microenvironment. This result suggests tumor cells are more likely colonized in more favorable microenvironment.

## Therapy Prospects for Lung Cancer Driver Gene

The discovery of various driver gene mutations has greatly promoted targeted therapies for lung cancer. According to the National Comprehensive Cancer Network guidelines, most targeted therapies recommended for NSCLC are those targeting *EGFR*, *ALK*, *ROS1*, *BRAF*, *RET*, and *MET* (148). Genetic testing has become one of the routine diagnostic procedures for patients after confirmation of NSCLC diagnosis. A single biopsy can capture most functionally important mutations in metastatic tumors, thereby providing necessary information for treatment decisions (149). NSCLC patients with positive driver genes have good sensitivity to TKIs. Prolonged survival has been achieved with radiotherapy combined with TKIs-targeted therapy in patients with brain metastases (150). Although good clinical effects can be achieved with first- and second-generation of TKIs, recurrent metastasis can occur, with the brain being the most frequent metastatic site. This may be a result of the blood-brain barrier to make the brain a tumor “refuge” (151). At present, improving penetration into the blood-brain barrier and intracranial activity is one of the key points in developing the third-generation TKI and new drugs. The development of nano-targeted drug systems might also benefit patients with brain metastases (152).

With the in-depth study of the mechanism for driver genes in lung cancer with brain metastasis, driver gene-associated signaling pathways, such as RAS/RAF, PI3K/AKT/mTOR, WNT/β-catenin, and JAK/STAT, also provide new targets for the treatment of lung cancer with brain metastases. mTORC1/2 inhibitor, for example, have demonstrated inhibition effects on tumor growth, EMT, metastasis, and improvements in anti-tumor immunity in preclinical models of lung cancer (153). JAK1/2 inhibitors also have potential therapeutic effects in patients with *KRAS* mutations (86). The PI3K signaling pathway is also enriched in brain metastases, suggesting an association of this pathway with increased risk of brain metastasis, which is expected to become a new therapeutic target (66, 154). However, none of these inhibitors has been studied in brain metastasis models. **Table 2** summarizes the potential biomarkers in lung cancer driver genes and lists the targeted drugs in brain metastasis. It is important to note that although targeted drugs of the rare driver gene associated with brain metastasis have not been studied in lung cancer, they have shown good results in other models of brain metastasis, such as breast cancer and melanoma (155, 156).

Moreover, the occurrence of secondary mutations in driver genes or other new mutations increases the complexity of the tumor genome, leading to drug resistance in targeted therapies and limiting patient’s survival (157). For NSCLC, more than 30% of patients with mutant *EGFR* undergo disease exacerbation due to brain metastasis during TKIs treatment (158, 159). On the other hand, the response to treatment is different between patients with or without driver gene mutation. For example, programmed death 1 (PD-1) inhibitor has played a role in treatment of NSCLC brain metastasis (160, 161). For patients without driver gene mutation, Ma ZY et al. found that the outcomes of patients with NSCLC presenting brain metastasis were comparable to patients without BMs when treated with nivolumab (PD-1 inhibitor) (162). This result suggests that driver genes are significant for the hierarchical management of patient treatment.

## Outlook

This review summarizes for the first time the signaling pathways related to driver genes and the role of these signaling pathways in the mechanism of brain metastasis. Tumor driver gene-associated signaling pathways are important signals for lung cancer with brain metastasis, which promotes tumor cell survival, invasion, and colonization. Furthermore, various cytokines and chemokine signals can be released after interaction of tumor cells with original resident cells in the brain or lung. These signals promote metastasis by driving gene-related signaling pathways. However, it is worthy of attention to researchers that in recent studies, lung cancer driver genes are related to brain metastasis and can be used as biomarkers for predicting brain metastasis, but there is still a lack of molecular mechanisms of brain metastasis starting with driver genes.

With the popularization of genetic testing technology, when patients with non-small cell lung cancer are diagnosed, clarifying the driver mutation status or intratumoral heterogeneity of the primary lesion can not only guide medication, but also predict the subsequent development of the tumor, including brain metastasis. Currently, only clinicopathologic variables, such as patient age, disease stage, and tumor histology, are used to predict the risk of brain metastasis (163–165). In future studies, common and druggable lung cancer driver genes, which have been confirmed to predict brain metastasis, can be combined with high-risk clinical features through artificial intelligence algorithms to establish a brain metastasis prediction model. For patients at high risk of brain metastasis, more treatment strategies, including PCI, targeted therapy, and immunotherapy, can be chosen. And high-risk patients without neurological symptoms also need regular computed tomography or magnetic resonance imaging. New lung cancer driver gene, which involved in pathways associated with brain metastasis, can be studied as potential biomarkers. Last but not the least, understanding the molecular characteristics of primary tumor and brain metastases can provide more information about tumor driver genes in the clinic for precise treatment.

## Author Contributions

YK performed a literature search, interpreted the data, and wrote the manuscript. YJ, QL, and XY supervised and contributed to the writing process. All authors contributed to the article and approved the submitted version.

## Funding

This work was supported by the National Natural Science Foundation of China (Grant no. 81773360).

## Conflict of Interest

The authors declare that the research was conducted in the absence of any commercial or financial relationships that could be construed as a potential conflict of interest.
